# Potency Physically Considered

**Published:** 1881-09

**Authors:** C. F. Millspaugh

**Affiliations:** Binghamton, N. Y.


### POTENCY PHYSICALLY CONSIDERED.

C. F. MILLSPAUGH, M. D., BINGHAMTON, N. Y.

THE vexatious question of the utility of high potencies is one that needs a thorough explanation before these great remedial agents will be generally used by the profession at large.

No true follower of the immortal teachings of HAHNEMANN doubts for a moment their efficacy; yet, when asked upon what he bases his faith in these thus potentiated drugs, he is unable to give a satisfactory answer, because he cannot realize, and becomes therefore incompetent to explain, the force that is inherent in these, to him, phenomenal preparations.

I purpose, in so far as lies in my power, to explain this force, and, in doing so, ground my reasonings upon the following proven law: *Every elemental atom, compound molecule, mass or part of a mass, organism or cell, has a molecular energy of a certain specific kind, distinctive, definite and absolute; a force perhaps allied, but never duplicated, in different substances, and that this force is the only true and radical distinction between such substances.*

All molecules are permissible, and, when in a state of mixture, act upon each other; one mode or rate of motion inevitably affecting another to a greater or less extent, and, as a result, that motion which has the greatest energy will change the motion of the others, until ultimately they partake of its identity, or will form a distinct or new substance, the motion of which will bear no similitude to any of its originators; *e. g.*, if equal volumes of hydrogen and chlorine, each gaseous, elemental and invisible, be mixed, and a flame applied, an explosion occurs, and, with the moisture of the air, a new substance—hydrochloric acid—is formed, having the properties of none of the original elements, and, according to our law, having therefore a molecular energy wholly its own.

Again, a portion of musk—that powerfully odorous substance from the musk-deer—*Moschus moschiferous*—has been allowed to perfume a room for forty years, and when weighed, after the lapse of that time, was found to have lost no portion of its mass, yet the room was always fully impregnated with its odor. What was it that was appreciable to the sense of smell? It was simply the surrounding air-molecules, which had gained the energy of the musk, and were so identical with it. This surrounding air would ever have remained musk had it not been for the preponderance of the air-molecules, which, by their force, from quantity, again revert the odorous molecules to their original motions, *i.e.*, those of air.

The great phenomena, heat, light, sound, electricity, etc., have been proven to be merely different modes of molecular motion, and are also proven to be transmutable. It is here that we may form some idea of the energy and rapidity of these motions, and of the different identities, whose only but fundamental specificalness is due to the mode, the rate, or the character of the motions of their molecules. I believe that we may account for infection by specific invisible miasms, for vitality, for disease, for death, for in fact every phenomenon present, by attributing to them this energy.

SAMUEL HAHNEMANN, by one of those master strokes which so characterize great genius, discovered that a drug, when potentiated, became of incalculable worth as a remedy for those symptoms of disease caused by that drug in the healthy, and so presented to a misguided profession the only true and scientific method of applying drugs to the cure of diseased conditions.

The force present in the thus potentiated drugs he called *dynamic power*, and the lamented HERING, in his honor, termed this force *Hahnemannism.* Either of these are good, but they explain nothing. What this force really is has been a hard-studied subject for over seventy years; its students being the while fully cognizant that no drug existed in any of such potencies above, at most, the 15th, thus found a phenomenon ready at their hands, and were accordingly puzzled and doubtful.

DR. RAU (*Werth der Homœopathische*, 134) says: Were the process of trituration and succussion attended by an actual increase of potency, it is evident that the drug would be rendered more and more unfit for homœopathic uses; and adds: the reverse is the case, and we must look upon the process as diminishing the power. Some years after, when he seemed fully to realize the mire into which he had fallen, he commences to seek more solid footing, and getting well out of this erroneous line of reasoning, he arrives at the conclusion that we potentiate to more minutely divide the crude drug, and so hasten its absorption, which was, indeed, a far better idea, and one more readily acceptable.

We do potentiate in part to more minutely divide the crude substance, but we do cure with "alcohol," when the drug is all gone! simply because the so-called "alcohol" has gained the energy of the drug, which cannot be lost, and is no longer alcohol, as such, but in reality the drug which was potentiated in it. This, too, notwithstanding DR. GREISSELICH, who says (*Handbuch*, 208): All notions of a transference of medicinal power to water, sugar-of-milk or alcohol, is a mysticism, unproven and unprovable.

KORSAKOFF believes that all the original substance disappears, or its material division ceases at the 6th centesimal trituration, and that thereafter the medicinal power is communicated to the vehicle by a process analogous to infection. What that infection is, we explain.

Precipitated tin, the most divisible of metals, has been seen under a microscope's objective in the 15th centesimal potency, and the particles were in a constant state of rapid and unvarying motion. PROF. HUXLEY saw in the vessels of a hair of the nettle (*Urtica urens*), the protoplasmic granules in a constant state of motion. It may be from these facts that an anonymous writer in the *Allgemeine Homœopathische Zeitung*, xxvii, 265, bases his opinion when he alleges that trituration produces in the substances so treated a lively molecular motion, which he calls *vivification of drugs*, or names the process such. Here is a writer whom I think came nearer the truth than he thought, had it only occurred to him to say *developed* instead of *produced*, and followed up his reasoning, he need not have remained so in obscurity.

The molecules composing a mass of any substance are, according to our law, in a constant and rapid state of motion, but being in juxtaposition, their motion, like that of a man in a crowd of his fellow-beings, is impeded; but isolate the molecule, or the man, and each will move as their power allows them.

This is the one use of the lower potencies. Now taking it for granted, until science shall have proven it, that the motions of water molecules, and those of alcohol and milk-sugar, are not so powerful *as those of any substance which retains its power when immersed in them,* and taking a drop of the last potency that contains drug, place it in not too great a number of drops of one of these vehicles, and what takes place? According to the postulates well-known to physicists, that motion begets motion, and that motion tends to be perpetuated in the absence of interfering causes, and calling attention to our solution of the so-called mystery of emanating scent, we see that it thereby gains the energy of the drug, and the energy of the drug being all its specific identity, the vehicle is now really the drug in a greatly diminished proportion, but a highly active state.

In that process called health, each cell of the organism has a molecular force peculiar to the function it performs, and vitality itself is simply the sum of these different energies. As long as these motions can hold their identity against all interfering energies, just so long are we healthy; but when any of these functional motions are changed by any disturbing cause, we immediately show symptoms of that force, or an entirely different disorder. The greater the disturbing force, the more acute the symptoms, even to death, which is really a total want of correlation of the molecular forces of life—nothing more.

To show how our potencies work, when applied in diseased conditions, let us take, as an illustration, a patient suffering under a change of his normal cell energies, caused by contact with those of scarlet fever, which had a greater energy, as witnessed by the attack, for had his forces been the stronger they would have resisted the disease; this fact is proven and self evident.

If his forces are allowed to remain the weaker, they will be entirely overcome. How shall we reinforce his wasted power? Simply by giving him that energy which is known to be antagonistic to his symptoms of energy lost. This force we will, in this case, call *Atropa belladonna.* How is the battle waged? The energy of the belladonna reinforces those warred against, and now by their greater power resist, and then by their preponderance again revert the cell motions to healthy activity, or the motions of health.

Of any other diseased condition, I see no cause why the result should not be equally happy, providing we are not too late, or that we have a knowledge of the proper force to use. And further assert that no disease has been, nor ever can be cured, except in two ways: the one by removing the counter force—*surgery, stimulants, hygiene, etc.*—and the other by the proper, or, in other words, homœopathic remedy. And that it is only through the higher potencies that we can produce the best results.—SCIRE FACIAS.

Read before the BINGHAMPTON HOMŒOPATHIC MEDICAL ASSOCIATION, July 21st, 1881, when discussing the question—*High Potencies, why do they act? and how?*

